# A Combined Method for Diabetes Mellitus Diagnosis Using Deep Learning, Singular Value Decomposition, and Self-Organizing Map Approaches

**DOI:** 10.3390/diagnostics13101821

**Published:** 2023-05-22

**Authors:** Mehrbakhsh Nilashi, Rabab Ali Abumalloh, Sultan Alyami, Abdullah Alghamdi, Mesfer Alrizq

**Affiliations:** 1 UCSI Graduate Business School, UCSI University, No. 1 Jalan Menara Gading, UCSI Heights, Cheras, Kuala Lumpur 56000, Malaysia; 2 Centre for Global Sustainability Studies (CGSS), Universiti Sains Malaysia (USM), George Town 11800, Malaysia; 3 Department of Computer Science and Engineering, Qatar University, Doha 2713, Qatar; 4 Computer Science Department, College of Computer Science and Information Systems, Najran University, Najran 55461, Saudi Arabia; 5 Information Systems Department, College of Computer Science and Information Systems, Najran University, Najran 55461, Saudi Arabia

**Keywords:** Singular Value Decomposition, diabetes, Self-Organizing Map, diagnosis, accuracy, deep learning

## Abstract

Diabetes in humans is a rapidly expanding chronic disease and a major crisis in modern societies. The classification of diabetics is a challenging and important procedure that allows the interpretation of diabetic data and diagnosis. Missing values in datasets can impact the prediction accuracy of the methods for the diagnosis. Due to this, a variety of machine learning techniques has been studied in the past. This research has developed a new method using machine learning techniques for diabetes risk prediction. The method was developed through the use of clustering and prediction learning techniques. The method uses Singular Value Decomposition for missing value predictions, a Self-Organizing Map for clustering the data, STEPDISC for feature selection, and an ensemble of Deep Belief Network classifiers for diabetes mellitus prediction. The performance of the proposed method is compared with the previous prediction methods developed by machine learning techniques. The results reveal that the deployed method can accurately predict diabetes mellitus for a set of real-world datasets.

## 1. Introduction

Diabetes, also known as diabetes mellitus, in humans is a rapidly expanding chronic disease [1,2] and has major impacts on modern societies [3]. As a person with diabetes mellitus cannot process food properly, glucose builds up in the bloodstream [4,5]. With diabetes, the body cannot produce sufficient insulin (type 1 diabetes) or cannot effectively utilize the hormone (type 2 diabetes) [6,7,8]. The body stops insulin in type 1 diabetes. In type 2 diabetes, the body’s cells do not respond effectively to insulin. This is due to the body’s immune system attacking and destroying a part of the pancreas mistakenly. Young people are usually affected by type 1 diabetes, mostly under 30 years of age. Type 2 diabetes, in contrast, often impacts middle-aged and older-aged people and is not fully curable. Some risk factors for type 2 diabetes are family history, age, ethnicity, being obese, having a diagnosis of gestational diabetes, high blood pressure, and obstructive sleep apnea.

The use of machine learning in disease diagnosis has been effective [9,10,11,12,13,14,15,16,17,18,19,20]. Many algorithms have been developed using supervised and unsupervised learning techniques and the deployment of real-world datasets for disease prediction [21,22]. Different machine learning techniques have been investigated for diabetes diagnosis in previous studies [23,24]. Although machine learning techniques have presented a powerful performance in knowledge extraction from diabetes medical datasets, the datasets are usually correlation-structured, non-normal, nonlinear, and complex. Therefore, the classification of diabetes disease is a challenging task. Furthermore, the missing values in datasets can influence the prediction accuracy of the deployed method. This has been a critical issue in the datasets for diabetes risk prediction. This study accordingly aims to develop a new combined method for diabetes mellitus diagnosis using deep learning, dimensionality reduction, and clustering approaches. The method is developed using a Self-Organizing Map (SOM), Singular Value Decomposition (SVD), and Deep Belief Network (DBN). We use SOM for data clustering and SVD for dimensionality reduction and missing value prediction. SOM is a competitive, cooperative, and well-known technique for describing the inherent connection between input and output [25]. To find the most important features, a feature selection method is applied to each cluster of SOM. SVD is a robust linear transformation for reducing dimensionality. It enables the space to be arranged to represent the main associative characteristics of the information while ignoring the smaller, less significant influences. The SVD transformation also produces zero mean and intercorrelated features [26]. DBN is used for diabetes mellitus prediction from a set of real-world diabetes mellitus data. DBN is a class of artificial intelligence (AI) computational techniques that enable an algorithm to program itself by learning from a large set of examples, eliminating the necessity to clearly define rules manually. It is powered by developments in computation and large amounts of data [27].

This research is organized as follows. In Section 2, we present related works. In Section 3, the methodology is provided. In Section 4, we introduce the dataset. In Section 5, we perform the data analysis. In Section 6, the discussion of the results is presented. In Section 7, we conclude this work. A list of abbreviations utilized in this research is presented in Table A1 of Appendix A.

## 2. Literature Review

In medical diagnosis research, disease classification has played a significant part in the medication development of various diseases [28]. Several studies have been conducted on the Diabetes Disease (DD) data classification by utilizing the PID dataset [19,29,30,31]. By deploying the dataset from the University of California, Irvine (UCI), ML warehouse, scholars have developed multiple approaches for overcoming DD diagnosis issues and enhancing the accuracy values. In DD classification research, various classification models and taxonomies have been presented in recent years, leading to a complex mix of detailed and imprecise terminologies. In the following, we will present a summary of previous studies that are present in this field.

Early diagnosis of retinal abnormalities has the chance to avoid diabetes, in addition to being vital for avoiding the development of diabetic retinopathy and/or diabetic macular edema (DME), as well as other abnormalities that could result in visual impairment. Existing DR screening techniques rely on retinal fundus imaging and conventional evaluation. Even so, such strategies are exceedingly laborious and expensive, as highly qualified experts are required for evaluation purposes [32].

In the study presented by Polat et al. [33], the authors designed a new approach for DD detection based on the GDA and the LS-SVM techniques. A novel cascade learning system was presented based on the above-mentioned techniques. The designed system entailed two stages: (1) GDA, which was used to preprocess the initial data and discriminate between normal and diabetes cases, and (2) LS-SVM, which was utilized in the second stage to categorize the diabetes datasets. The outcomes presented a classification accuracy of 82.05%, which was considered an encouraging outcome when compared with prior findings reported by other classification techniques.

A study by Aslam et al. [34] used GP for diabetes classification. In this study, new features were produced using combinations of the current features of the disease, without proceeding knowledge of the probability distribution. The designed method had multiple basic steps. First of all, the features were selected and utilized to generate an ordered set of initial features. By referring to the ordered set, features were added one after another to constitute several subsets of the initial features. Following that, new features were produced from each subgroup of initial features using GP-CPS to utilize the strengths and weaknesses of GP-generated features. In the final step, the performance of the produced features was evaluated using the K-NN and SVM techniques. The outcomes of the study compared to other approaches presented a superior performance. A new approach was introduced to classify medical records by Kahramanli and Allahverdi [35]. In addition, a hybrid NN system, which entailed the ANN method and FNN approach, was designed. Two real-time datasets were utilized for checking the suitability of the proposed approach. In order to assess the performance of the suggested approach, several metrics were used. The proposed approach achieved ACC values of 84.24% and 86.8% for the two datasets. The proposed method presented the best outcome compared with proceeding outcomes obtained from similar previous studies.

A new method was presented by Erkaymaz and Ozer [36] for DD diagnosis based on the SW-FFANN approach, which was the first study that deployed this approach for DD. The authors developed a small-world network by following the Watts–Strogatz method, utilizing the proposed structure for DD classification, and contrasting the research outcomes with regular or conventional FFANN. The authors indicated that SW-FFANN presented better outcomes than the conventional FFANN in terms of output correlation, output error parameters, and classifier performance. When compared with other approaches that utilized the Pima Indians Diabetes database, the deployed approach presented a better performance.

Fuzzy methods have become one of the prominent solutions for classification issues, as they can enhance the interpretability of the outcomes and present more insight into the classifier scheme and decision-making procedure. A study by Ganji and Abadeh [37] used a system to extract a group of fuzzy conditions for DD classification based on the ACO technique. The new system had novel features that distinguish it from other similar systems. The classification accuracy was 84.24%, which indicated that the proposed system outperformed multiple well-known systems. Another study that deployed fuzzy methods was presented by Dogantekin et al. [38]. An intelligent system for DD diagnosis was developed based on the LDA and ANFIS techniques by Dogantekin et al. [38]. The architecture of the DD diagnosis system contained two main components: LDA, which was utilized to extract feature variables between healthy and DD patient data. Four main metrics were used in the evaluation of the system: SE and SP, ACC, and CM. The results indicated that the classification accuracy of the system was around 84.61%. A comparative Pima-DD diagnosis was accomplished by Temurtas et al. [39]. For this purpose, a MLNN architecture, which was trained by the LM algorithm, and a PNN architecture were utilized. The outcomes of the study were assessed compared to the outcomes of prior reports concentrating on the DD diagnosis and utilizing the same ML database. The classification accuracy of MLNN with LM obtained by the study using correct training was better than those obtained by other studies for the conventional validation method. The outcomes, based on PID, that resulted from using PNN architecture, presented a good performance compared with the outcomes from other research, especially for similar approaches. However, the study presented far less classification accuracy (10× FC) (66.78%) compared to the study by [40]. Another study by Hayashi and Yukita [29] utilized the PID dataset by incorporating a rule extraction algorithm and sampling selection techniques to achieve high-quality results in terms of ACC and conciseness. The ACC value was 83.83% by using the rule extraction algorithm, while the sampling selection technique presented better results in terms of the ACC value and conciseness. The novelty of this study is reflected by its ability to overcome the black box model by adopting the rule extraction method to present such explanations.

SVM was used by many DD diagnosis researchers [19,41]. In a study by Çalişir and Doğantekin [41], a DD diagnosis system based on LDA and MWSVM was presented. The architecture of the proposed system entailed three steps: LDA was used in the feature extraction and feature reduction stage; MWSVM was used in the classification step; and the third step entailed the evaluation process of the result based on the following metrics: SE, SP, ACC, and CM. The ACC of the proposed method was obtained at about 89.74%. Compared to previous studies that used the same approach, it presented a better performance, such as [33] presented an ACC of 78.21% and [33] presented an ACC of 79.16%.

Another study by Nilashi et al. [19] developed a DD diagnosis system based on ML techniques, particularly the EM, PCA, and SVM approaches. EM was used basically for clustering, PCA was used for removing the noise, and SVM was used for performing the classification. The incremental PCA, along with the incremental SVM, were used for the incremental learning of data. The experimental findings on the PID dataset presented an enhancement of the ACC value compared to the conventional methods. The study stressed the importance of the deployment of incremental learning on additional datasets to enhance the computation time. Aiming to improve the DD diagnosis accuracy, Sisodia and Sisodia [30] used three ML techniques: DT, NB, and SVM. Experiments were conducted on the PID dataset. The assessment of the research outcomes was conducted, focusing on several metrics of PR, ACC, F-Measure, and REC. The ACC value was measured based on correctly and incorrectly classified instances. The findings indicated that, among the inspected algorithms, NB had the highest accuracy value of 76.30%. Still, the study is limited in terms of the assessment of the presented results compared to other related works. NN approaches have been utilized in many studies regarding DD diagnosis [31,35,36]. ML approaches were utilized in a study presented by Nilashi et al. [31] by designing a DD classification system that combined the SOM, PCA, and NN techniques. SOM was used for clustering, PCA was used for removing the noise, and NN was used for the DD diagnosis. The experimental outcomes on the PID dataset presented an enhancement of the accuracy over previous reports in the same context. The deployed approach helped in enhancing the accuracy significantly compared to other approaches, such as general regression neural networks by [42], GDA–LS-SVM by [33], and SW-FFANN by [36].

Another study that adopted the NN approach was presented by Edla and Cheruku [43]. The study adopted RBFNN and utilized a Bat-based clustering algorithm. The designed approach was evaluated on the PID dataset and presented outcomes that surpass other variants of RBFNN in several studies, such as the Dunn index-RBFNN by [44] and Bee-RBF model by [45]. Another study presented by Khan et al. [46] aimed to classify the DD medical status among patients using ANN approaches, particularly the LM algorithm on the PID dataset. The result of the study was assessed based on measures of SE and SP for various learning rules. The study indicated that the LM algorithm surpassed other approaches in terms of the considered evaluation measures. It also helped in addressing the convergence issue in some other algorithms. In a study by Maniruzzaman et al. [47], the GC approach was used to overcome the non-normality, inherent correlation structure, and nonlinearity issues. The outcomes were assessed compared to the current methods, such as NB and LDA, according to several metrics of ACC, SE, SP, PPV, NPV, and ROC, and presented encouraging outcomes compared to other techniques that were deployed in the literature, such as ANN and AIS by [48] and SVM by [49]. DL techniques were used by many studies in the context of DD classification [28,50]. In a study by Kannadasan et al. [28], a framework based on DL was designed for DD classification. Several metrics were used for the assessment of the designed system, and an overall ACC value of 86.26% resulted. In a work presented by Alhassan et al. [50], a huge dataset for DD was presented (KAIMRCD). The dataset entailed comprehensive information about 14,000 cases. Aiming to achieve an accurate T2DM diagnosis, the dataset was processed as a time series and then explored through a TP-DL approach. Additionally, LSTM and GRU were trained on the dataset and presented a high ACC value of over 97%.

PK-SVM was used by Longato et al. [51] to differentiate between individuals impacted by IGT vs. T2D based on CGM-mined GV instances and focus on several essential variables: age, sex, and BMI. The outcomes presented an acceptable ACC value of 87.1%. In a study by Rahman et al. [24], a novel DD classifying model based on Conv-LSTM was developed. In addition, three well-known approaches: CNN, T-LSTM, and CNN-LSTM were assessed, and the outcomes were compared against the outcome of the proposed model using the PID dataset. The evaluation findings revealed that Conv-LSTM presented the best performance among the assessed approaches, with the highest ACC value of 97.26%. Devi et al. [52] presented a combined method that utilized the FF clustering technique and SMO technique for classifying DM. To achieve the research goal, the FF algorithm was used to segment the data into multiple segments. As the dataset size reduced, the result of the time used for the computation was minimized noticeably. The output of the FF technique was presented to the SVM, which categorized the cases into diabetic and non-diabetic, based on the PID and achieved a high ACC value (99.4%). In a study by Bhatia et al. [23], an effective home-based supervision system for DD classification was presented. The system entailed four basic layers: DDA, DDC, DME, and DPDM layers to classify, extract, predict, and classify DD. In addition, RNN was used for analyzing the prediction regarding the probabilistic quantification of UbD supervision considering the LoDI and DIM. Additionally, UbD existence was viewed based on the SOM process. The research outcomes were assessed based on several measures and presented enhanced values compared to other studies.

In summary, assessing the efficacy of DD classification is a complicated issue that should be tested on newer and more comprehensive diabetes datasets in future research, so that the most highly accurate rules can be extracted for diagnosis [29].

## 3. Methods

**Figure 1:**
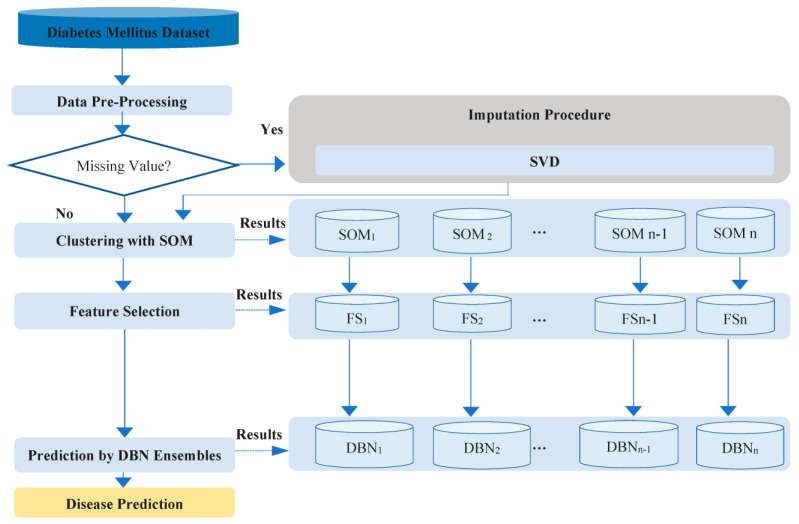
Research method.

The method of this study is developed for the diagnosis of diabetes mellitus. The general framework of the proposed method is presented in Figure 1. The method uses SVD for missing value prediction, SOM for clustering the data, STEPDISC for feature selection, and an ensemble of DBN classifiers for diabetes mellitus prediction. These techniques are introduced in the following sections. In the first step of the proposed methodology, the data is preprocessed. Then, the data clustering is performed. The clustering of SOM is performed with the aid of PCA, as the noise of the data may impact the classification accuracy. The aim of performing clustering is to provide more accurate classification results. In addition, the prediction model of segments that include diabetes mellitus is more effective than the model that considers the whole dataset for diabetes classification. In addition, in the proposed approach, we incorporate the feature selection strategy to find the most important features to predict diabetes mellitus by the use of ensembles of DBN classifiers.

### 3.1. SVD

In this study, SVD as a dimensionality reduction technique was used for the missing value prediction. The procedure for missing value prediction by SVD is presented in Algorithm 1. This method can predict the missing value in the datasets through five main steps. To do so, we first convert the data in each cluster to the dense matrix $B^{m,n}$. Then, we perform the normalization procedure $B^{m,n}$. In the third step, the SVD technique is applied to the matrix obtained in the second step. The obtained matrix Z is used for the approximation of Z, $Z_{d}$. SVD finally predicts the missing value in the last step.
**Algorithm 1:** Missing value prediction by SVD.**→Converting data to the dense matrix** 
$B^{m,n}$**.**
**→Normalization using**  
$Z_{ij}=\frac{B_{ij}-{\bar{B}}^{{}_{j}}}{\sigma_{j}}$
${\bar{B}}^{j}=\frac{1}{m}\sum_{i=1}^{m}B_{ij}$**,**
$\sigma_{j}^{2}=\frac{1}{m-1}\sum_{i=1}^{m}(B_{ij}-{\bar{B}}^{j}{)}^{2}$
**where**
${\bar{B}}^{{}_{j}}$
**
is the average value and
**
$\sigma_{j}$
**
indicate the SD for
**
$B^{{}_{j}}$**.**
**
→
**
**
SVD technique on Z is applied.
**

**→**$Z_{d}$
**
is computed for approximation of Z.
**
**
→
**
**
Missing value is calculated using
**
${\bar{B}}^{j}+\sigma_{j}{\left(Z_{d}\right)}_{ij}$**.**


### 3.2. SOM

Kohonen Self-Organizing Maps (SOM) [53] have been utilized as a robust technique for data clustering. The SOM structure includes one input and one output layer [54]. The objective of SOM is to transfer all input data objects with n dimensions to the output in a manner in which the objects are related to each other [55,56]. The SOM is able to perform unsupervised training for the datasets, in which the target is clustering the data [14,15,57,58,59,60,61]. The Euclidean distance [62,63] from the input vector to all nodes is computed when the input vector is given to the network. All weight vectors of the SOM network are randomly initialized, and from the training dataset, an input pattern X is chosen. Through the comparison of the Euclidean distances, the similarity to the input vector is found, and this will enable the network to identify the winning node, the Best Matching Unit (BMU), which is closest to the input vector. Three processes, which are competition, cooperation, and adaptation, are essential in the SOM algorithm. In the competition step, the winning neurons for observation are identified. In the cooperation process, SOM determines a topological neighborhood, and cooperation is ensured between nodes. In the adaptation process, according to the example, the synaptic weights are arranged for the neurons affected by competition.

### 3.3. DBN Technique

DBN was designed by Hinton et al. [64] to overcome the vanishing gradient issue presented in prior research [65]. DBN is a robust multilayered generative design that is constructed from pretrained layers, and it is categorized as a DNN approach [17]. DBN entails multiple RBMs that are composed of a Visible Layer (VL) and a Hidden Layer (HL); the first is considered as an input component, while the second is regarded as an output part. Fully linked nodes in different layers are presented without links among the nodes in the internal layers. RBM attempts to learn the distribution of the probability from VL to HL using an Energy Function (EF) [17]. DBN entails the pretraining step (unsupervised) of the deep RBMs and the fine-tuning step (supervised) of the classification layer. Following the prior training step, the standard backpropagation formula is utilized in a controlled style to refine all the weights to achieve a specific role. Weights initialization of the network using DBN is applied effectively in several domains related to artificial intelligence [66]. DBN presents a good feature extraction result; hence, it is suitable for detecting the features from the data. As DBN is a completely linked structure, it eases the analysis of the data more than any other DNN.

**Figure 2:**
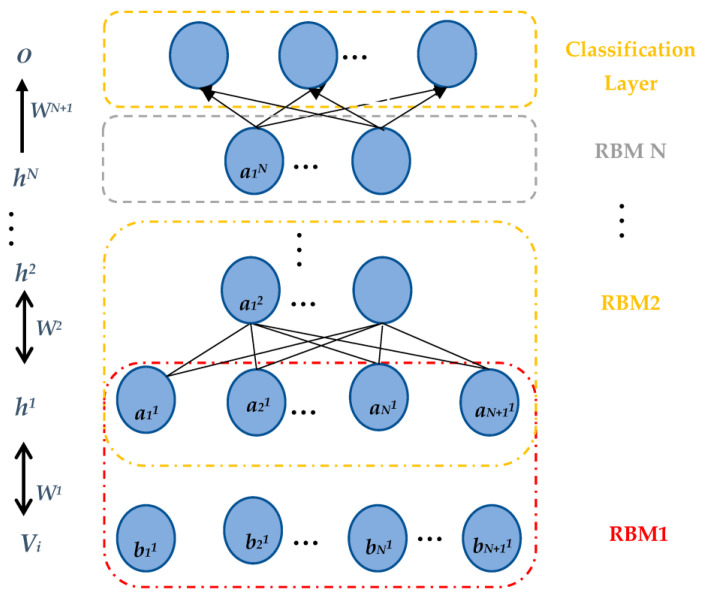
The DBN architecture.

As presented in Figure 2, each RBM is structured from a VL that entails the V-units, $v=\left\{v_{1},v_{2},\ldots ,v_{i}\right\},$ and a HL that entails the H-units, $h=\{h_{1},h_{2},\ldots ,h_{j}\}$.

Considering the model variables of DBN, θ=[W,b,a], the energy function can be presented as:

(1) $$E\left(v,h;\theta \right)=-\sum_{i=1}^{I}\sum_{j=1}^{J}w_{ij}v_{i}h_{j}-\sum_{i=1}^{I}b_{i}v_{i}-\sum_{j=1}^{J}a_{j}h_{j}$$

where $w_{ij}$ is the weight of the link among the visible part $v_{i}$, in which the total value is I, and hidden part h, which total value is J, and $b_{i}$ and $a_{j}$ indicate the bias requirements of the visible and hidden parts, respectively. The joint distribution over all the nodes is measured regarding the function of the energy, $E\left(v,h;\theta \right),$ as:

(2) $$p\left(v,h;\theta \right)=\frac{exp\left(-E\left(v,h;\theta \right)\right)}{Z}$$

where $Z=\sum_{h;v}exp\left(-E\left(v,h;\theta \right)\right)$ is the function of the partition. The conditional probabilities of the hidden and visible nodes h and v can be measured as:

(3) $$p\left(h_{i}=1|v;\theta \right)=\delta \left(\sum_{i=1}^{I}w_{ij}v_{i}+a_{j}\right)$$

(4) $$p\left(v_{i}=1|v;\theta \right)=\delta \left(\sum_{j=1}^{J}w_{ij}h_{i}+b_{i}\right)$$

whereby δ is a logistic equation, $\delta \left(x\right)=1/1+\exp\left(x\right)$.

The joint probability is optimized by training RBMs. Several RBMs are stacked to construct the DBN, whereby the output of the *l*th layer (H nodes) is utilized as the input of the I+1th layer (V nodes). The training procedure is indicated by basic steps: prior training and fine-tuning. In the prior training step, the input data are provided to the VL of the initial RBM and converted into the HL, which is continually processed in the following RBM. Afterward, the layer-to-layer unsupervised training is completed and the DBN learns the features from the given input, then the features are provided to the classifier layer of the DBN. Eventually, fine-tuning is completed on the classification layer to improve the DBN performance.

### 3.4. Featured Selection by STEPDISC

Based upon several quantitative variables and classification variables, a step-by-step discriminant analysis is carried out by the STEPDISC procedure to select a subset of the quantitative variables to be used in class discrimination [67]. The group of variables that comprise each class is supposed to be normally multivariate, with a common covariance matrix. Observations containing missing values are omitted from the analysis, which may impact the accuracy of the classification models. Forward and backward strategies are used in STEPDISC. In this research, the backward strategy is used. For the stopping rule, the significance level of the F-test from the covariance analysis is used.

## 4. Dataset

**Figure 3:**
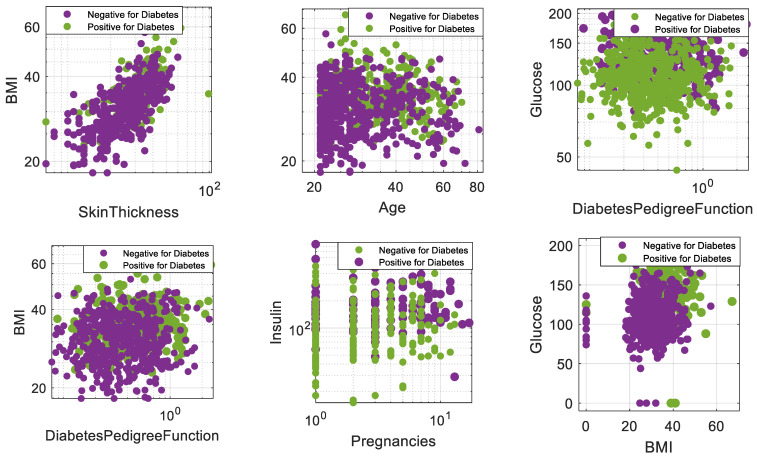
Visualization of the features of the diabetic dataset.

The diabetic dataset includes 768 female patients. It has 500 controls and 268 diabetic patients. This dataset was taken from the UCI (University of California, Irvine) in November 2019. The National Institute of Diabetes and Digestive and Kidney Diseases provided the original data for this dataset. The dataset’s goal is to diagnose whether or not a patient has diabetes based on certain diagnostic measurements included in the dataset. The patients are at least 21 years old of Pima Indian heritage. In this dataset, 374 of these patients have zero serum insulin levels, 27 of them have zero body mass indexes, 35 patients have zero diastolic blood pressure, the skinfold thickness is zero for 227 patients, and 5 patients have zero glucose levels. We consider these zero values as missing values. According to the WHO standards, these details can be used to detect the critical symptoms that can be used to predict diabetes in patients. The target variable is a binary variable indicating whether or not a patient has diabetes, with 1 indicating that the patient has diabetes and 0 indicating that the patient does not have diabetes. Demographics of the diabetic patient cohort are shown in Figure 3 and Table 1. This dataset has been widely used in research on diabetes prediction and has been used as a benchmark dataset for developing and evaluating machine learning algorithms for diabetes prediction.

## 5. Results and Methods Comparison

**Figure 4:**
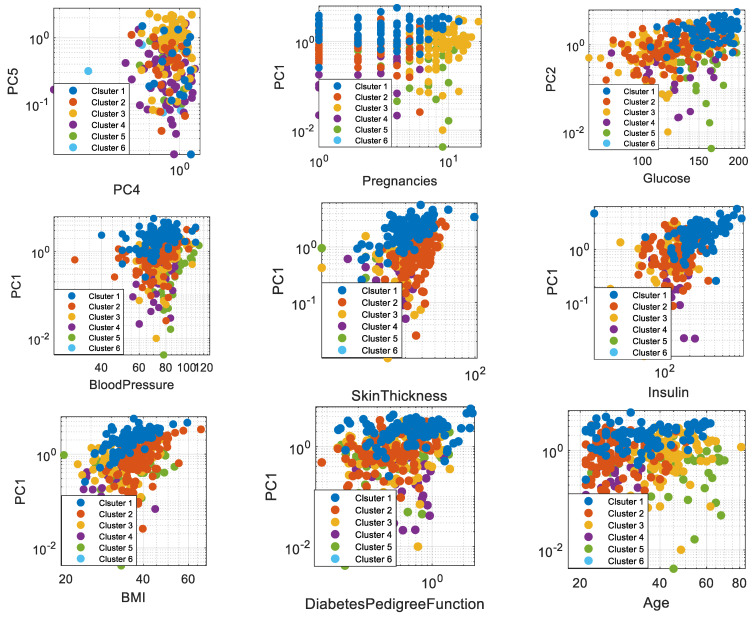
The results of SOM clustering.

**Figure 5:**
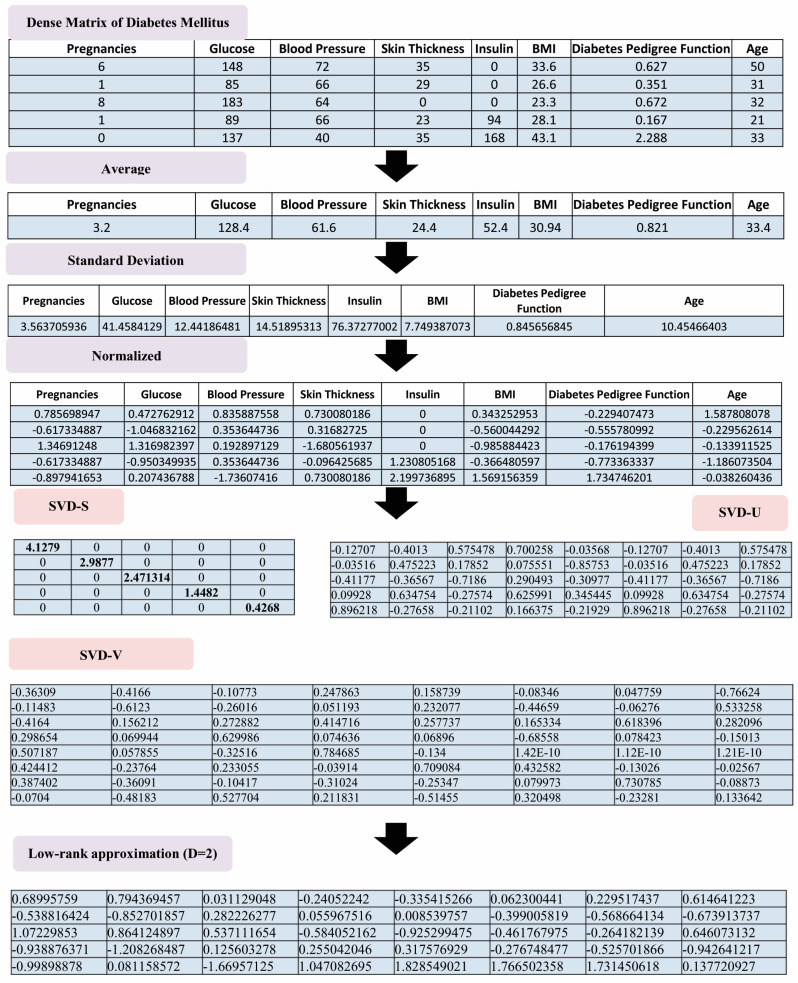
An example of performing the SVD algorithm for missing values prediction.

**Figure 6:**
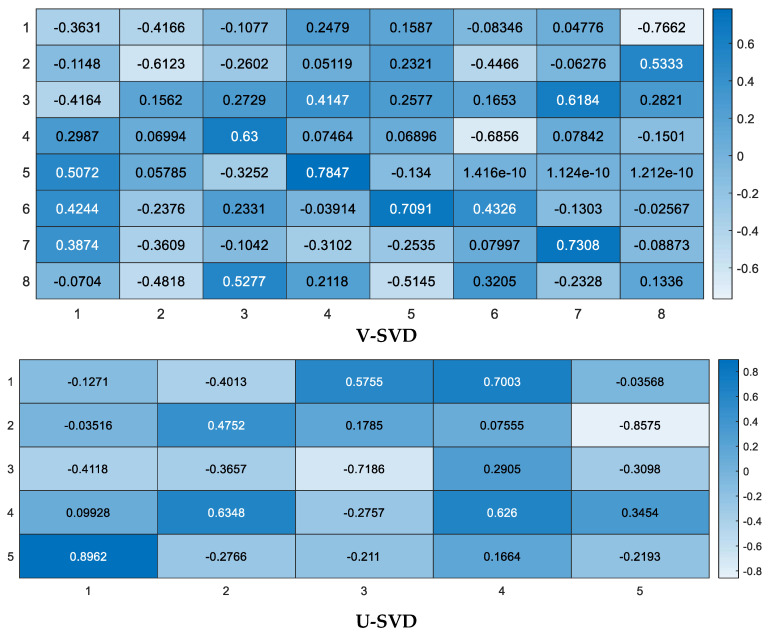
SVD results for diabetes mellitus diagnosis.

In the first step of the data analysis, we use SOM for data clustering. The results for SOM clustering are presented in Figure 4. The results are presented on different PCs of the PCA. Overall, six clusters were discovered by the SOM algorithm. In fact, SOM2×3 resulted in the best map quality for data clustering. The number of samples in Clusters 1–6 is, respectively, 93, 130, 104, 242, 132, and 67. They are visualized by PC4 and PC5, as shown in Figure 5. In this figure, pregnancies, glucose, blood pressure, skin thickness, insulin, BMI, diabetes pedigree function, and age are also visualized in different clusters through different PCs. After data clustering, we apply the SVD algorithm for the missing value prediction. An example of how the SVD algorithm can predict the missing values in the diabetic patient dataset is presented in Figure 5. The procedure is able to perform the prediction through the decomposition of matrices, which include the data of each cluster. The example is provided for low-rank approximation (D = 2) of decomposed matrices. The SVD result for diabetes mellitus diagnosis is presented in Figure 6.

**Figure 7:**
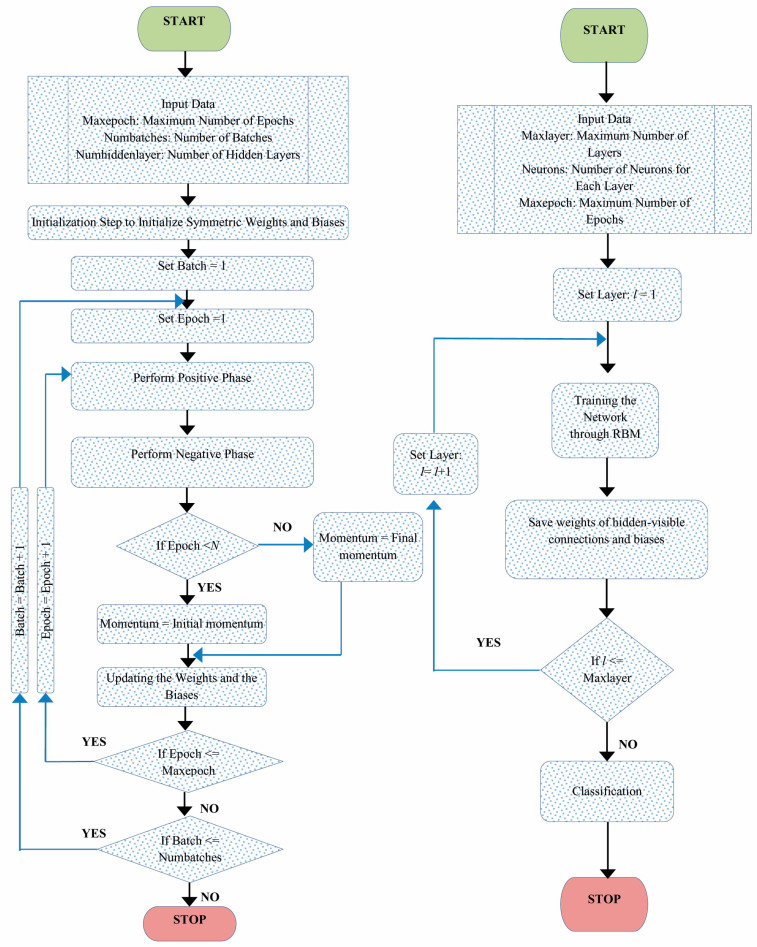
RBM iterative learning process and DBN training process.

In this paper, the classification models are constructed based on the DBN. In each cluster, the bottom of the model is stacked by RBM layers. In addition, the expected output variables are at the top of the model, which is the classification layer. To overcome the data processing complexity problem, one-class–one-network is adopted to improve the ability of the neural network. This approach was suggested by several researchers in different contexts, such as automatic handwriting recognition [68], speech recognition [69], and face recognition [70]. The global one-class–one-network aims to coordinate all subnetwork outputs in classification problems. This can present an appropriate choice for the tradeoff between accuracy and complexity. We applied DBN onto each cluster to construct the classification molds for diabetes mellitus diagnosis. The data for each cluster was divided into training and test sets. To avoid Gradient Diffusion (GD), we applied DBNs’ pretraining and fine-tuning procedures. Selecting an appropriate number of hidden layers and the number of their nodes is important in constructing DBN for the classification task. In this study, the number of hidden layers in DBN is fixed to two. To train the DBN to construct the models, the number of neurons in the input layer was set equally to the number of input variables in the dataset. The number of output layer neurons was set to the number of dataset classes equally. With a batch size of 100, the unsupervised training and fine-tuning algorithms were performed for 200 epochs in each run. In Figure 7, RBMs’ iterative learning process and DBN training process are presented.

**Figure 8:**
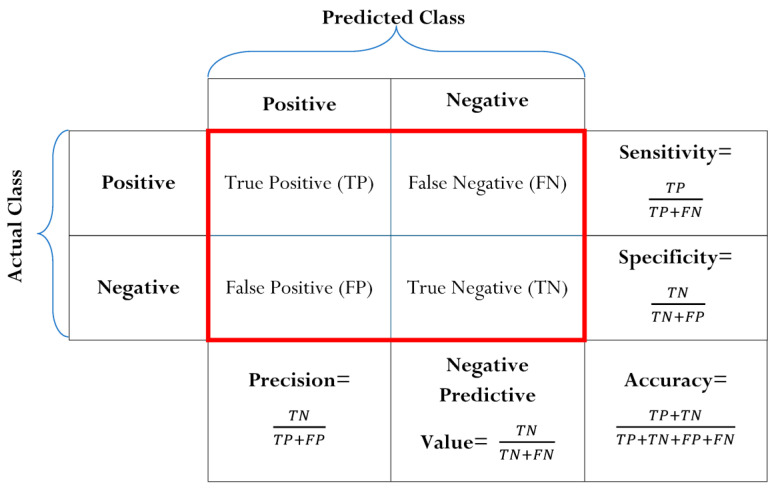
Confusion matrix.

The results of our method are evaluated through the accuracy metric. In Figure 8, the formulas to calculate the accuracy are presented. It is obtained through the confusion matrix, which includes True Positive (TP), False Negative (FN), False Positive (FP), and True Negative (TN).

**Figure 9:**
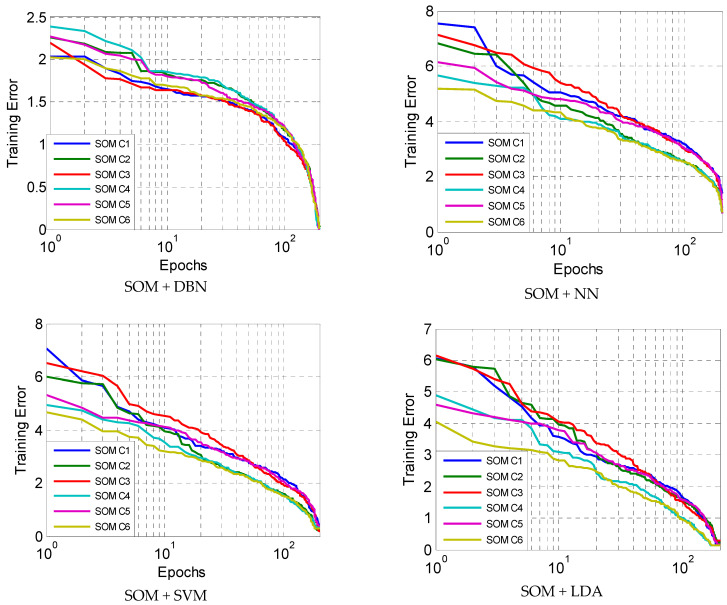
Methods comparison.

The proposed method is compared with the other classifiers combined with the SOM clustering. The training errors of all the classifiers, DBN, SVM, NN, and LDA, in six clusters are presented in Figure 9. The results are presented for 200 epochs. It is found that the proposed method, SOM + DBN, has better training errors with faster convergence in all clusters.

In addition, it can be seen from the results in Figure 9 that there is no fluctuation in all methods during the learning process, which means that the architecture provided by clustering and classifiers may be stable enough to learn an accurate classification model for the diabetes mellitus diagnosis. We further evaluated the methods by the accuracy metric. The results for all methods are provided in Table 2.

In addition, our method is compared with the General Regression Neural Network [42], GDA-LSSVM [33], MWSVM [41], and SW-FFANN [36]. The results show that the SOM + DBN ensembles approach provides the best accuracy results (98.32%) for diabetes mellitus classification in relation to the General Regression Neural Network [42] (80.21%), GDA-LSSVM [33] (79.16%), MWSVM [41] (89.74%), and SW-FFANN [36] (91.66%). Furthermore, it is found that the clustering method combined with the classifiers has achieved more accurate classification results.

## 6. Discussion

Diabetes is a significant disease, because it affects a large number of people and, if not properly managed, can lead to serious health complications [71]. Diabetes is a chronic disease that occurs when the body’s ability to regulate blood sugar levels is compromised. Diabetes is classified into two types: type 1 and type 2 [72]. Type 1 diabetes occurs when the body’s immune system attacks and destroys insulin-producing cells in the pancreas, whereas Type 2 diabetes occurs when the body becomes insulin-resistant or fails to produce enough insulin to regulate blood sugar levels. Diabetes can cause a variety of health problems, including cardiovascular disease, nerve damage, kidney damage, vision damage, and foot damage. It can also cause other health issues, such as high blood pressure, high cholesterol, and obesity.

Using state-of-the-art computational approaches to aid in complicated medical care choices has been indicated since the 1970s [73]. Still, medical approval and employment of these approaches have moved slower than intended [74]. This can be due to several causes that range from the restricted use of research outcomes to the limited computational performance of these approaches. Hence, with the availability of online medical datasets, it is critical to deploy efficient computational approaches in medical contexts such as disease diagnosis.

Designing new disease diagnosis approaches is a comprehensive process that entails the integration of theory, data gathering, and appropriate data analysis [75]. In the design of the disease diagnosis approach, a group of indicators that reflect the folds of disability resulting from the disease is essential [76]. Diabetes patients need to control and manage their health day by day, which can influence their daily activities. The single method for diabetes patients to tolerate DD is to maintain their blood sugar at normal levels, which can only be managed if the used treatment entails the appropriate diet, taking oral diabetes treatment or some kind of insulin, and practicing suitable exercises [77]. Additionally, the medication for DD is very complex and costly for medical centers [31]. As there are several significant aspects to document regarding the DD that can aid health staff in reaching the appropriate choice about treatment, utilizing the appropriate method for the disease diagnosis is of great importance.

ML addresses the advancement of techniques that enable machines to learn. The obstacle that faces researchers in this regard is to design algorithms that can gather a set of patterns and present new presumptions from the primary data without the need for human intervention. For ML, classification concerns dividing a group of data into multiple classes, referring to the training outcomes of a subgroup of data that have a predefined classification. Clustering is a procedure that entails dividing a group of observations into multiple reasonable segments based on specific metrics of resemblance among each segment. The clustering approach has been utilized in several disease diagnosis systems [19,78,79,80], which indicates its wide utility as one of the procedures in experimental medical data analyses.

As a particular fold of ML, DL techniques have been used for exploring various medical datasets to classify diseases and predict their effects [81,82]. DL classification systems have achieved multiple aims in several medical contexts, such as image and unstructured data processing and to aid healthcare staff in their decisions [17]. Many DL systems have accomplished high accuracies at various diagnostic functions among multiple medical conditions [83,84,85]. The performance of the DL approach highly relies on the optimum measures of variables. The optimum measures of variables are achieved by tuning with various measures and observing the performance where the approach presents a better accuracy. The main aim of this research is to design a new approach for DM diagnosis by combining several techniques and enhancing the disease prediction accuracy. The main contribution of this research is the design of a new approach that incorporates dimensionality reduction, clustering, feature selection, and DBN ensemble techniques to enhance the accuracy prediction of the diabetes risk. The advantage of the current approach can be indicated by the best accuracy outcome compared to other techniques that used the SOM and DBN techniques using the UCI dataset. To summarize, the outcomes of our research on a UCI dataset indicated the efficiency of combining the dimensionality reduction, clustering, and DBN techniques in enhancing the diagnosis accuracy of the diabetes risk.

## 7. Conclusions

ML has been effectively deployed in the diagnosis of diabetes. This study presented a novel integrated approach using machine learning techniques for early-stage diabetes risk prediction. SVD, SOM, STEPDISC, and DBN ensemble techniques were integrated to develop this method. SVD was used for dimensionality reduction and missing value prediction. SOM was used to cluster the data. STEPDISC was used for feature selection. DBN ensemble was used for diabetes mellitus prediction. To assess the proposed approach, multiple experimental analyses were performed on a real-world dataset taken from UCI. The deployed approach was assessed compared to other approaches in the literature and the methods combined with the SOM clustering. For 200 epochs, the proposed approach, SOM+DBN, presented better training errors, with rapid convergence in all clusters. In addition, the outcomes of the evaluation of the approaches were compared in terms of the accuracy metric and presented an overall accuracy of 98.32% when using the SOM and DBN ensamples.

The encouraging outcomes of this study can be utilized practically by physicians to help them reach the right diagnosis. The research outcomes can be also deployed by scholars to explore the deployed methods on other datasets and in other medical contexts. In the context of medical disease diagnosis, there will always be room for enhancing the performance of classification models. This is particularly important in medical contexts, as the outcomes will impact human lives.

There are still plenty of future directions for researchers on dimensionality reduction, clustering, and DBN approaches for diabetes risk prediction to utilize all their advantages. Future research can deploy the same method in other contexts in the health field. The research can be also extended to assess the performance of the applied techniques on other datasets, particularly large datasets. More studies should explore this point in the design of new approaches to face the difficulties of data computation time and to benefit from big data effectively. The research on DD classification can be carried out by focusing on different stages of clustering, noise removal, and classification. Future research can deploy different methods that adopt incremental learning that adjusts the model based on the new cases. Research can follow other routes that address some issues in the datasets, such as data sparsity.

## Figures and Tables

**Table 1:** Demographics of the diabetic patient cohort.

SN	Attributes	Descriptions	Attributes Type	Mean ± SD
1	Pregnant	Number of times pregnant	Continuous	3.84 ± 3.36
2	Glucose	Plasma glucose (2 h)	Continuous	121.67 ± 30.46
3	Pressure	Diastolic blood pressure (mm Hg)	Continuous	72.38 ± 12.10
4	Triceps	Triceps skin fold thickness (mm)	Continuous	29.08 ± 8.89
5	Insulin	Two hours serum-insulin (μ U/mL)	Continuous	141.76 ± 89.10
6	Mass	Body mass index (weight in kg/(height in m)^2^)	Continuous	32.43 ± 6.88
7	Pedigree	Diabetes pedigree function	Continuous	0.47 ± 0.33
8	Age	Age (years)	Continuous	33.24 ± 11.76
9	Class	Diabetic (500) vs. Control (268)	Categorical	-

**Table 2:** Comparison of the proposed method with other classifiers for DM (Diagnosis Methods) comparison.

Method	Reference	Accuracy
General Regression Neural Network	[42]	80.21%
GDA-LSSVM	[33]	79.16%
MWSVM	[41]	89.74%
SW-FFANN	[36]	91.66%
SOM + SVM	This study	90.73%
SOM + LDA	This study	91.83%
SOM + NN	This study	84.62%
SOM + DBN Ensembles	This study	98.32%

## Data Availability

The data are available in the UCI machine learning archive.

## Appendix A

**Table A1:** List of abbreviations utilized in this research.

Abbreviation	Description
ACC	Accuracy
ANN	Artificial Neural Network
ACO	Ant Colony Optimization
ANFIS	Adaptive Network Based Fuzzy Inference System
AIS	Artificial immune systems
CGM	Continuous Glucose Monitoring
BMI	Body Mass Index
CM	Confusion Matrix
CNN	Convolutional Neural Network
CNN-LSTM	Convolutional Neural Network Long Short-term Memory
Conv-LSTM	Convolutional Long Short-term Memory
DBN	Deep Belief Network
DD	Diabetes Disease
DDA	Diabetic Data Acquisition
DDC	Diabetic Data Classification
DIM	Diabetes Infection Measure
DM	Diabetes Mellitus
DME	Diabetic Mining and Extraction
DPDM	Diabetic Prediction and Decision Making
DT	Decision Trees
SD	Standard Deviation
FF	Farthest First
FNN	Fuzzy Neural Network
GC	Gaussian Classification
GDA	Discriminant Analysis
GP	Genetic Programming
GP-CPS	GP with comparative partner selection
IGT	Impaired Glucose Tolerance
K-NN	K-Nearest Neighbor
KAIMRCD	King Abdullah International Research Centre Diabetes
LDA–MWSVM	Morlet Wavelet Support Vector Machine Classifier
LDA	Linear Discriminant Analysis
LM	Levenberg–Marquardt
LoDI	Level of Diabetic Infection
LS-SVM	Least Squares Support Vector Machine
ML	Machine Learning
ML	Machine Learning
ML	Expectation Maximization,
MLNN	Multilayer Neural Network
NB	Naive Bayes
NN	Neural Network
NPV	Negative Predictive Value
PCA	Principle Component Analysis
PID	Pima Indian Diabetes
PK-SVM	Polynomial Kernel-Support Vector Machine
PNN	Probabilistic Neural Network
PPV	Positive Predictive Value
PR	Precision
ROC	Receiver Operating Characteristic
REC	Recall
RBFNN	Radial Basis Function Neural Networks
RNN	Recurrent Neural Network
SE	Sensitivity
SMO	Sequential Minimal Optimization
SOM	Self-Organizing Map
SP	Specificity
SVM	Support Vector Machine
SW-FFANN	Small-World Feed Forward Artificial Neural Network
TP-DL	Temporal Predictive Deep Learning
T2D	Type 2 Diabetes
T-LSTM	Traditional Long Short-term Memory
UbD	Urine-based Diabetes
